# Supplementary material for: Prognostic biomarkers of intracerebral hemorrhage identified using targeted proteomics and machine learning algorithms
Source: PLoS One. 2024 Jun 3;19(6):e0296616. doi: 10.1371/journal.pone.0296616 (PMC11146689; doi:10.1371/journal.pone.0296616)
Supplement: S1 Table — (DOCX) [file pone.0296616.s001.docx]

# **S1 Table. List of shortlisted peptides of proteins for MRM and the role of these proteins in ICH pathophysiology.**

| **S. No** | **Protein Name** | **Peptide Sequence** | **Q1 m/z** | **Q3 m/z** | **Protein description** | **Role in ICH** |
| --- | --- | --- | --- | --- | --- | --- |
| 1. | Alpha-2-macroglobulin | AIGYLNTGYQR | 628.3251 | 1071.522  738.3529  851.437  624.31  523.2623  1014.5 | A large plasma glycoprotein, functions as a broad-spectrum proteinase inhibitor. Synthesized in the liver. | Not defined. The proposed role may relate to its function in modulating thrombin’s proteolytic activity leading to ICH-induced brain injury. |
|  |  | NEDSLVFVQTDK | 697.8435 | 737.3828  836.4512  1151.594  1036.567  949.5353 |  |  |
| 2. | Apolipoprotein A-I (APO-A1) | QGLLPVLESFK | 615.8583 | 819.4611  932.5451  623.3399  722.4083  1102.651 | A predominant component of high-density lipoprotein and plays a crucial part in lipid metabolism. Synthesized in the liver and small intestine. | APO A1 is not a direct participant in the processes leading to ICH. Its role in maintaining cardiovascular health and reducing inflammation can indirectly influence the risk factors associated with ICH. |
|  |  | VSFLSALEEYTK | 693.8612 | 940.4622  853.4302  782.3931  1053.546  669.309  540.2664 |  |  |
|  |  | DYVSQFEGSALGK | 700.8383 | 1023.511  808.4199  532.3089  661.3515  1122.579  936.4785 |  |  |
| 3. | Apolipoprotein L1 (APO-L1) | VNEPSILEMSR | 637.82 | 522.2341  635.3181  748.402  932.487  1061.53 | A component of HDL and is involved in lipid and glucose metabolism. Expressed in the liver, vasculature, and kidneys. | Not defined. APO-L1’s indirect role in ICH pathophysiology may be explained by its role in chronic inflammation and endothelial dysfunction and  the association of its genetic variants with higher risk of hypertension-related kidney disease. |
| 4. | Alpha-1-acid glycoprotein 2 (ORM2) | SDVMYTDWK | 572.7526 | 843.3706  712.3301  942.439  1057.466  697.2862 | An acute-phase inflammatory protein synthesized in the liver and extrahepatic sites. Blood levels significantly elevated during acute inflammation. | Not defined. Its indirect role in ICH may be attributed to immunomodulatory properties, binding and transport of molecules released due to hemorrhage, and acute phase response to ICH during inflammation. |
|  |  | EHVAHLLFLR | 412.24 | 548.3555  435.271  661.4396  574.2732  366.1772 |  |  |
| 5. | Serum amyloid P-component (APCS) | AYSLFSYNTQGR | 703.8386 | 972.4534  825.385  1172.569  1085.537  738.3529 | A nonfibrillar glycoprotein present in all human amyloid deposits and synthesized in the liver. | Not defined. It may indirectly influence the clearance of cellular debris and metabolites in ICH. |
| 6. | Apolipoprotein C-I (APO-C1) | TPDVSSALDK | 516.764 | 834.4203  620.325  719.3934  931.4731  533.293 | The smallest protein component of all apolipoproteins that is synthesized in liver. It connects with triglyceride-rich lipoproteins and HDL. | Not defined. APO-C1’s effects on lipid metabolism and cardiovascular health can indirectly influence the risk factors for ICH. |
|  |  | MREWFSETFQK | 496.9029 | 739.3621  603.2708  652.3301  750.3392  523.2875 |  |  |
| 7. | Ig kappa chain V-I region Ni | ASNLETGVPSR | 565.7937 | 745.3839  616.3413  858.468  972.5109  673.3151 | Primarily related to the variable region of immunoglobulin molecules; plays a crucial role in the immune system by binding to specific antigens and initiating immune responses. | No well-established direct role in the pathogenesis of ICH. |
| 8. | 72 kDa type IV collagenase (MMP2) | VDAAFNWSK | 519.2562 | 823.4097  938.4367  752.3726  681.3355  534.2671 | Also known as a 72kDa type IV collagenase and Gelatinase A and belongs to a family of proteolytic enzymes known as matrix metalloproteinases. | Involved in blood-brain barrier disruption and inflammatory response after ICH. |
| 9. | Multiple inositol polyphosphate phosphatase 1 (MINPP-1) | NATALYHVEAFK | 682.3539 | 730.3883  893.4516  1006.536  1077.573  1178.62 | It hydrolyzes inositol pentakisphosphate and inositol hexakisphosphate metabolites. Its deficiency leads to imbalance in inositol polyphosphate metabolism inside the cell. | Not defined with limited research on MINPP-1s involvement in ICH. |
| 10. | Serpin A11 | SLLHTLALPSPK | 426.259 | 612.3715  541.3344  826.5033  736.4352  849.5193  826.5033 | A member of the serine protease inhibitor (serpin) family. Plays a role in regulating proteases, particularly serine proteases. | Not defined with limited research on ICH. |
| 11. | F-box/WD repeat-containing protein 5 (FBXW5) | TVMVAD**C[CAM]**SR [Carbamidomethyl] | 519.739 | 838.3546  608.2457  707.3141  422.1816  201.1234 | A DNA damage binding WD40 protein that functions as a substrate recognition component of the E3 ubiquitin-protein ligase complexes. | Not defined. It may play roles in various cellular processes and responses to stress or injury. |
| 12. | Serotransferrin (TF) | MYLGYEYVTAIR | 739.871 | 1071.547  1184.631  851.4621  1014.525  722.4196 | A plasma glycoprotein that binds to free iron in the bloodstream and transports it into cells in a reversible manner. It is the main protein that regulates iron hemostasis. | Involved in iron regulation, and body’s response to injury and recovery in ICH patients. |
|  |  | EGYYGYTGAFR | 642.2882 | 771.3784  934.4417  714.357  1097.505  551.2936 |  |  |
|  |  | HSTIFENLANK | 637.3304 | 1136.595  1049.563  829.3839  835.4308  1013.505 |  |  |
| 13. | Haptoglobin (HP) | VTSIQDWVQK | 602.322 | 1003.521  803.4046  1104.568  675.3461  916.4887 | Found in the liver and binds to haemoglobin, thereby prevents any iron loss or kidney damage. It is an antioxidant with antibacterial properties and regulates several facets of the acute phase response. | Haptoglobin plays cytoprotective roles and is a potential therapeutic target for ICH treatment. |
|  |  | TEGDGVYTLNNEK | 720.3361 | 881.4363  1209.575  1037.526  1152.553  980.5047  504.2413  617.3253 |  |  |
|  |  | VGYVSGWGR | 490.7511 | 562.2732  881.4264  661.3416  563.2824  824.405 |  |  |
| 14. | Beta-2-glycoprotein 1 (APOH) | PDDLPFSTVVPLK | 714.3927 | 987.5873  357.2496  456.318  743.4662  890.5346  656.4341 | A soluble blood protein with its primary functions including regulation of complement and haemostasis. | Not defined. It’s role in lipid metabolism, coagulation and the production of hypertension may relate to ICH. |
|  |  | VYKPSAGNNSLYR | 490.2563 | 538.2984  451.2663  1078.528  652.3413  1061.501  981.4748 |  |  |
|  |  | V**C**[CAM]PFAGILENGAVR  [Cysteine Carbomedomethylated] | 751.8928 | 1243.679  928.5211  999.5582  758.4155  645.3315  516.2889 |  |  |
| 15. | Plasma protease C1 inhibitor (SERPING1) | GVTSVSQIFHSPDLAIR | 609.6635 | 908.4948  771.4359  684.4039  1055.563  835.9441 | A serine protease inhibitor that plays a significant role in regulating the complement and contact systems of the immune response. | Not defined. It may indirectly influence aspects of inflammation and coagulation in ICH. |
|  |  | LLDSLPSDTR | 558.7984 | 575.2784  890.4214  775.3945  557.2678  1003.505  688.3624 |  |  |
| 16. | Retinol-binding protein 4 (RBP4) | YWGVASFLQK | 599.8164 | 849.4829  693.393  1035.562  622.3559  792.4614 | A member of the lipocalin family predominantly expressed in the liver. It is the main transport protein for the hydrophobic molecule retinol, (vitamin A) in the circulatory system. | Not defined. Serum levels of RBP-4 are associated with blood pressure that may relate to ICH. |
|  |  | DPNGLPPEAQK | 583.296 | 669.3566  572.3039  384.1514  346.2085  839.4621 |  |  |
| 17. | Insulin-like growth factor-binding protein 3 (IGFBP3) | FLNVLSPR | 473.2795 | 685.3991  472.2878  571.3562  359.2037  798.4832  375.2027 | One of the six members of the IGFBP family and plays a crucial role in the IGF pathway and is the most abundant protein in blood circulation. | Not defined. IGFBP-3 is not typically considered a direct factor in the development or pathophysiology of ICH. |
|  |  | EMEDTLNHLK | 615.2952 | 606.2076  969.5  840.4574  725.4304  624.3828  511.2987 |  |  |
|  |  | AGASSAGLGPVVR | 571.3198 | 527.33  697.4355  942.5367  470.3085  615.3097  768.4726 |  |  |
| 18. | Glial Fibrillary Acidic Protein (GFAP) | HLQEYQDLLNVK | 500.5999 | 701.4192  473.3082  360.2241  812.4512  586.3923 | A cytoskeletal protein that is mostly found in astrocytes. | Higher GFAP levels lead to abrupt rupture of the blood-brain barrier and brain injury in ICH patients. |
|  |  | LEAENNLAAYR | 632.32 | 1021.506  821.4264  950.469  480.2565  707.3855  593.3406 |  |  |
|  |  | FADLTDAAAR | 525.7644 | 832.4159  604.3049  717.389  903.453  503.2572  388.2303 |  |  |
| 19. | Brain Natriuretic Peptide (BNP) | ISSSSGLGCK | 498.2449 | 882.3986  795.3665  708.3345  621.3025  364.1649 | A vasoactive or neurohormone synthesized in the cardiac tissue that has natriuretic, diuretic, and vasodilator functions. | A prognostic indicator in ICH and an effective marker of disease severity. |
|  |  | MVLYTLR | 448.2445 | 665.3802  389.2329  288.1852  764.4487  552.2962 |  |  |
| 20. | Matrix metalloprotenase-9 (MMP-9) | AFALWSAVTPLTFTR | 840.959 | 1092.605  835.4672  734.4196  934.5356  403.234  290.1499 | A collagenase that cleaves types IV and V collagen protein. | A marker of brain injury. Plays a key role in extracellular matrix destruction and blood brain barrier disruption in ICH patients. |
|  |  | QLSLPETGELDSATLK | 851.4489 | 1260.632  1034.536  933.4888  242.1499  329.1819  519.3137 |  |  |
|  |  | AVIDDAFAR | 489.2562 | 464.2616  579.2885  694.3155  807.3995  171.1128 |  |  |
| 21. | D-dimer | QGFGNVATNTDGK | 654.8126 | 319.1612  420.2089  635.2995  706.3366  805.405  976.4694 | A breakdown product of fibrin mesh that suggests thrombus development following factor XIII stabilisation. | A marker of early neurological deterioration and poor outcome in ICH patients. |
|  |  | YLQEIYNSNNQK | 757.3677 | 534.2558  590.2893  704.3322  867.3955  980.4796  1109.522 |  |  |
| 22. | Ubiquitin carboxyl-terminal hydrolase isozyme L1 (UCH-L1) | LGVAGQWR | 443.7483 | 546.2783  617.3154  716.3838  773.4053  270.1812  171.1128 | A cytoplasmic deubiquitinating enzyme that is abundant in neurons and neuroendocrine cells. It plays a potent role in maintaining synaptic plasticity and the brain’s self-repair mechanisms post-injury. | Not defined. Probable role may indicate a marker of neuronal damage or injury in ICH patients. |

**Abbreviation**: m/z- mass to charge ratio.
