# Supplementary material for: Prognostic biomarkers of intracerebral hemorrhage identified using targeted proteomics and machine learning algorithms
Source: PLoS One. 2024 Jun 3;19(6):e0296616. doi: 10.1371/journal.pone.0296616 (PMC11146689; doi:10.1371/journal.pone.0296616)
Supplement: S2 Table — (DOCX) [file pone.0296616.s002.docx]

# **S2 Table. Gradient table for MRM proteomics.**

| **Time (minutes)** | **% buffer A** | **% buffer B** |
| --- | --- | --- |
| 0.0 | 95.0 | 5.0 |
| 2.0 | 95.0 | 5.0 |
| 15.0 | 70.0 | 30.0 |
| 19.0 | 50.0 | 50.0 |
| 23.0 | 20.0 | 80.0 |
| 25.0 | 10.0 | 90.0 |
| 27.0 | 95.0 | 5.0 |
| 30.0 | 95.0 | 5.0 |
