# Supplementary material for: Prognostic biomarkers of intracerebral hemorrhage identified using targeted proteomics and machine learning algorithms
Source: PLoS One. 2024 Jun 3;19(6):e0296616. doi: 10.1371/journal.pone.0296616 (PMC11146689; doi:10.1371/journal.pone.0296616)
Supplement: S3 Table — (DOCX) [file pone.0296616.s003.docx]

# **S3 Table. The prognostic potential of protein biomarkers in predicting 90-day and 180-day poor outcome after intracerebral hemorrhage.**

| **S. No** | **Protein Biomarker (UniProt ID)** | **Cutoff** | **OR (95% CI)** | **p-value** | **Sensitivity (95% CI)** | **Specificity (95% CI)** | **PPV (95% CI)** | **NPV (95% CI)** |
| --- | --- | --- | --- | --- | --- | --- | --- | --- |
| **90-day poor outcome in ICH** | | | | | | | | |
| 1 | UCH-L1 (P09936) | <13.85 | 2.94 (1.38-6.27) | 0.005 | 74% (64-82%) | 51% (35-68%) | 81% (72-88%) | 41% (27-56%) |
| 2 | Haptoglobin (P00738) | >16.65 | 3.87 (1.44-10.42) | 0.007 | 92% (85-96%) | 26% (13-42%) | 78% (70-84%) | 53% (29-76%) |
| 3 | Alpha-2-Macroglobulin (P01023) | >17.87 | 2.24 (1.00-5.00) | 0.04 | 80% (71-87%) | 36% (21-53%) | 78% (69-85%) | 39% (23-56%) |
| 4 | Serpin A11 (Q86U17) | >11.36 | 3.22 (1.05-9.87) | 0.04 | 94% (87-97%) | 18% (8-34%) | 53% (49-57%) | 74% (51-88%) |
| **180-day poor outcome in ICH** | | | | | | | | |
| 1 | UCH-L1 (P09936) | <14.03 | 2.46 (1.15-5.23) | 0.02 | 78% (69-86%) | 40% (26-56%) | 73% (63-81%) | 47% (31-64%) |
| 2 | Alpha-2-Macroglobulin (P01023) | >17.84 | 2.43 (1.09-5.40) | 0.03 | 82% (73-89%) | 34% (21-49%) | 72% (63-80%) | 48% (31-66%) |
| 3 | MINPP1 (Q9UNW1) | >19.08 | 3.01 (1.10-8.22) | 0.03 | 92% (84-96%) | 21% (11-36%) | 71% (62-78%) | 56% (31-78%) |
| 4 | MMP2 (P08253) | >18.72 | 2.17 (1.05-4.46) | 0.04 | 71% (61-80%) | 47% (32-62%) | 73% (63-82%) | 44% (30-59%) |

The cut-off values represent the Log_2_ normalized protein concentrations.

**Abbreviations**: OR: Odds Ratio; CI: Confidence Interval; PPV: Positive Predictive Value; NPV: Negative Predictive Value; UCH-L1: Ubiquitin C-Terminal Hydrolase L1; MINPP1: Multiple inositol polyphosphate phosphatase 1; MMP2: Matrix Metalloproteinase 2 (72 kDa type IV collagenase).
