# Supplementary material for: Prognostic biomarkers of intracerebral hemorrhage identified using targeted proteomics and machine learning algorithms
Source: PLoS One. 2024 Jun 3;19(6):e0296616. doi: 10.1371/journal.pone.0296616 (PMC11146689; doi:10.1371/journal.pone.0296616)
Supplement: S4 Table — (DOCX) [file pone.0296616.s004.docx]

# **S4 Table. The prognostic potential of protein biomarkers in predicting 90-day and 180-day mortality after intracerebral hemorrhage.**

| **S. No** | **Protein Biomarker (UniProt ID)** | **Cutoff** | **HR (95% CI)** | **p-value** | **Sensitivity (95% CI)** | **Specificity (95% CI)** | **PPV (95% CI)** | **NPV (95% CI)** |
| --- | --- | --- | --- | --- | --- | --- | --- | --- |
| **90-day mortality in ICH** | | | | | | | | |
| 1 | FBXW5 (N=86) (Q969U6) | >8.78 | 2.28 (1.22-4.30) | 0.01 | 39% (24-55%) | 89% (76-96%) | 76% (53-92%) | 61% (49-73%) |
| 2 | APO-C1 (P02654) | <16.91 | 1.77 (1.04-2.99) | 0.03 | 66% (53-78%) | 53% (42-64%) | 58% (51-65%) | 61% (51-70%) |
| 3 | IGFBP3 (P17936) | <16.48 | 1.81 (1.09-3.00) | 0.02 | 60% (46-72%) | 60% (49-70%) | 51% (39-63%) | 67% (56-78%) |
| 4 | UCH-L1 (P09936) | <13.85 | 2.13 (1.15-3.92) | 0.02 | 79% (67-88%) | 41% (31-52%) | 49% (39-59%) | 73% (59-85%) |
| **180-day mortality in ICH** | | | | | | | | |
| 1 | FBXW5 (Q969U6) | >8.80 | 2.02 (1.08-3.79) | 0.03 | 34% (20-49%) | 86% (71-95%) | 75% (51-91%) | 52% (39-65%) |
| 2 | MMP9 (P14780) | <15.99 | 1.81 (1.11-2.95) | 0.02 | 60% (47-71%) | 65% (53-75%) | 60% (47-71%) | 65% (53-75%) |
| 3 | UCH-L1 (P09936) | <13.85 | 1.99 (1.12-3.53) | 0.02 | 78% (66-87%) | 42% (30-53%) | 54% (43-64%) | 68% (53-81%) |
| 4 | APO-C1 (P02654) | <17.00 | 1.72 (1.02-2.88) | 0.04 | 69% (56-79%) | 48% (36-60%) | 53% (42-64%) | 64% (50-76%) |

The cut-off values represent the Log_2_ normalized protein concentrations.

**Abbreviations**: HR: Hazard Ratio; CI: Confidence Interval; PPV: Positive Predictive Value; NPV: Negative Predictive Value; APO- Apolipoprotein; IGFBP3: Insulin-like growth factor-binding protein 3; FBXW5: F-box/WD repeat-containing protein 5; UCH-L1: Ubiquitin C-Terminal Hydrolase L1; MMP- Matrix Metalloproteinase.
