# Supplementary material for: Prognostic biomarkers of intracerebral hemorrhage identified using targeted proteomics and machine learning algorithms
Source: PLoS One. 2024 Jun 3;19(6):e0296616. doi: 10.1371/journal.pone.0296616 (PMC11146689; doi:10.1371/journal.pone.0296616)
Supplement: S5 Table — (DOCX) [file pone.0296616.s005.docx]

# **S5 Table. Functional enrichment analysis of protein biomarkers that can predict poor outcome and mortality after ICH.**

| **Category** | **Description** | **Background Genes** | **Genes** | **P-value** | **FDR value** | **Genes** | **Term Name** |
| --- | --- | --- | --- | --- | --- | --- | --- |
| GO Biological Process | Negative regulation of catalytic activity | 807 | 6 | 9.17E-07 | 0.0118 | UCHL1, A2M, SERPINA11, HP, MMP9, APOC1 | GO:0043086 |
| GO Cellular Component | Extracellular space | 3195 | 8 | 1.68E-05 | 0.0285 | MMP2, A2M, SERPINA11, HP, MINPP1, MMP9, IGFBP3, APOC1 | GO:0005615 |
| GO Biological Process | Extracellular matrix disassembly | 66 | 3 | 4.94E-06 | 0.0317 | MMP2, A2M, MMP9 | GO:0022617 |
| GO Biological Process | Protein metabolic process | 4251 | 9 | 8.75E-06 | 0.0327 | MMP2, UCHL1, FBXW5, A2M, HP, MINPP1, MMP9, IGFBP3, APOC1 | GO:0019538 |
